# Milk Therapy: Unexpected Uses for Human Breast Milk

**DOI:** 10.3390/nu11050944

**Published:** 2019-04-26

**Authors:** Malgorzata Witkowska-Zimny, Ewa Kamińska-El-Hassan, Edyta Wróbel

**Affiliations:** Department of Biophysics and Human Physiology, Medical University of Warsaw, Chalubinskiego 5, 02-004 Warsaw, Poland; malirob@poczta.onet.pl (E.K.-E.-H.); mwzgenetyka@onet.pl (E.W.)

**Keywords:** human milk benefits, colostrum, milk therapy, bioactive factors

## Abstract

Background: Human breast milk provides a child with complete nutrition but is also a popular therapeutic remedy that has been used in traditional, natural pharmacopeia, and ethnomedicine for many years. The aim of this current review is to summarize studies of non-nutritional uses of mothers’ milk. Methods: Two databases (PubMed and Google Scholar) were searched with a combination of twelve search terms. We selected articles that were published between 1 January 2010, and 1 January 2019. The language of publication was limited to English. Results: Fifteen studies were included in the systematic review. Ten of these were randomized controlled trials, one was a quasi-experimental study, two were in vitro studies, and four employed an animal research model. Conclusions: Many human milk components have shown promise in preclinical studies and are undergoing active clinical evaluation. The protective and treatment role of fresh breast milk is particularly important in areas where mothers and infants do not have ready access to medicine.

## 1. Introduction

Human breast milk (HBM) is perhaps the most important functional food known. It is a dynamic food with both nutritional and health benefits for neonates and infants. Human milk has powerful immunological properties, protecting infants from respiratory diseases, middle ear infections, and gastro-intestinal diseases. It is now appreciated that human breast milk has health impacts that are lifelong, with breastfeeding showing protective effects against diabetes mellitus, obesity, hyperlipidemia, hypertension, cardiovascular diseases, autoimmunity, and asthma [1]. However, human milk is also a popular therapeutic remedy that has been applied as a part of traditional, natural pharmacopeia, and ethnomedicine for many years. Public health nurses have reported on the effects of fresh colostrum and human milk as a treatment for conjunctivitis, chapped nipples, rhinitis, infections of the skin and soft tissues. The discovery of growth factors, cytokines, and a heterogeneous population of cells—including stem cells, probiotic bacteria, and the HAMLET complex (human alpha-lactalbumin made lethal to tumor cells)—in human milk has led to researchers’ increased interest in human breast milk as a natural medicine. In recent years, human milk has been the focus of many types of evidence-based research. There have been a number of reports on the topical application of human milk as an effective treatment for diaper rash, atopic eczema, diaper dermatitis, and umbilical cord separation [2,3,4]. The protective and treatment role of human milk is particularly important in areas where mothers and infants do not have ready access to medicine, such as in developing countries. In these situations, milk therapy is often a determining factor of infant recovery and survival. For this reason, more clinical trials and research into mothers’ milk come from low and middle-income countries in Africa and Asia. Many human milk components have shown promise in preclinical studies and are undergoing active clinical evaluation. A few milk-derived therapeutic preparations are available to clinicians. The study of human milk has resulted in abundant opportunities for translational medicine. However, complementary and alternative medicine (CAM) therapies often fare unfavorably under the scrutiny of evidence-based practice (EBP), due to the lack of or shortage of research and the inherent differences in healing ideology. Yet excess medicalization and pharmaceuticalization can lead to the extension of the medical gaze to human conditions. Physically maladaptive outliers have been treated as diseases and pulled into the realm of medicine. Many “non-disease” states can creep up into medicine, and with time become medicalized due to the redefining of many conditions that were long considered social or psychological phenomena as disease states. Processes regarded as natural are now looked at medical problems or diseases. Unnecessary medicalization leads to great social and financial cost, as well as increased anxiety and the risk for complication from further workups for incidental or clinically unimportant findings. The growth of research and reflections on medicalization has led to the proposal of other parallel concepts, such as biomedicalization. These tools could be useful in the analysis of human enhancement and can be defined as contributing to a “bionic society”. Medicalization risks neglecting the role of social determinants, natural therapies, and ethnomedicine in shaping human health. On the other hand, where individuals do not have ready access to medicine—particularly in developing countries—knowledge, skills, and practices based on the theories and experiences indigenous to different cultures, whether explicable or not, are used in the maintenance of health as well as in the prevention, improvement, and treatment of illness.

There is no doubt that incorporating traditional and modern evidence-based medicine (EBM) as integral parts of the formal health-care system is important and likely to be achieved in many countries. The aim of the current review is to summarize studies of non-nutritional usage of mothers’ milk. Due to its low cost, wide availability, and lack of undesirable effects, mothers’ milk has the potential to play a role in human health and in evidence-based therapy.

## 2. Methods

The literature review was performed by conducting an electronic search of PubMed and Google Scholar. No filter or limitation was used during the search. Reference lists from selected studies were manually scanned to identify any other relevant studies. The electronic search used the following keywords and medical subject headings (MeSH) terms: Human milk; breast milk, mother milk, colostrum, atopic eczema, diaper dermatitis, nipple pain, breast inflammation, umbilical cord separation, neonatal conjunctivitis, HAMLET, topical treatment. The two authors independently searched databases and reviewed articles. Bibliographic references to retrieved reviews and studies were searched for additional articles. We included articles that were published between 1 January 2010, and 1 January 2019. The language of publication was limited to English.

**Figure 1:**
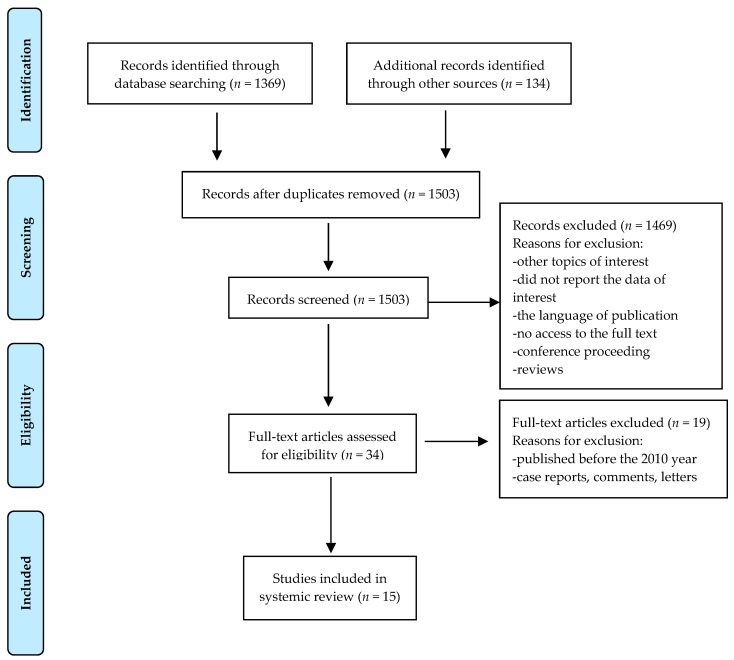
Flow diagram.

From total of 1503 initially screened articles, only 15 fulfilled all the inclusion criteria. The following criteria needed to be fulfilled for a study to be included in this review: (1) Topical application of human milk or human milk active factors versus control; (2) in the case of human studies, participants had to be newborns; (3) in the case of animal models, HBM in vitro assessment had to be included. Articles that did not provide sufficient information from the title and abstract were included for further evaluation, and reading was done in full. Records were first selected after which 1469 were excluded based on excluded criteria: Did not report the data of interest, the language of publication, no access to the full text, conference proceeding, reviews. There was no restriction of study designs included. In order to limit bias in the inclusion–exclusion process, the selection was made on the basis of the consensus of two authors. The Cohen’s kappa index was calculated to assess the agreement between the two reviewers and any discrepancy was resolved by consensus or by a third reviewer. The reviewers were not blind to author, institutions, or manuscript journals. Data extraction and analysis were performed by the same two reviewers. Figure 1 presents a flow diagram of the review process while Table 1 summarizes the studies that were included.

We present a narrative summary of studies, rather than a meta-analysis, because of the heterogeneity in measurement tools, populations, interventions, and design (whether qualitative or observational). The reporting in this review follows the Preferred Reporting Items for Systematic Reviews and MetaAnalysis (PRISMA) guidelines, where warranted.

## 3. Results

Fifteen studies were of sufficient quality to be included in the systematic review. All of these had been published in peer-reviewed journals. The agreement between the reviewers was substantial: κ = 0.625 (*p* = 0.02). Seven studies were conducted in Iran, two in the USA, two in each of Turkey and Sweden, and one in each of Norway and Germany (Table 1).

Ten of these fifteen studies were randomized controlled trials (two on experimental mice models), and one was a quasi-experimental study. We also considered two in vitro studies and four with animal research models. We decided to include these because they have practical implication for clinical trials and, in our opinion, are examples of translational science.

Below we briefly describe the studies by medical problem.

### 3.1. Skin Problems: Atopic Eczema and Diaper Dermatitis

Recently, a few studies have been published on the topical anti-inflammatory effects of human breast milk in the treatment of skin problems, such as atopic eczema and diaper dermatitis [6,20,21]. Our systematic review included five randomized clinical trials devoted to infants’ skin problems, but the results were not consistent.

Berents and colleagues, in a small pilot study, did not find any effect on eczema spots treated with topical application of fresh human milk. However, this clinical trial has some limitations. First, it had a very small study population of six children; second, two of them were treated with their mother’s milk produced for a younger sibling. The mean age of the children was 18.5 months (ranging from 4 to 32) [5].

Kasrae et al. randomized 104 Iranian infants with atopic dermatitis for 21 days of treatment with 1% hydrocortisone versus human milk. The frequency of healed infants was 81.5% and 76% in the human milk and 1% hydrocortisone groups on day 21, respectively. The findings suggest that human milk can improve atopic eczema with similar results and is as easy to apply as 1% hydrocortisone ointment (*p* < 0.001), but without the side-effects and cost [6].

The effects of topical application of human milk and 1% hydrocortisone were also compared in the treatment of diaper dermatitis [7]. The randomized group consisted of 141 infants (aged 0–24 months). Parents received general advice about diaper rash care and were instructed to apply the medication for seven days. The mothers assigned to use milk were asked to rub milk gently on the affected area at the end of each breastfeeding session. Hydrocortisone 1% in an ointment base was applied sparingly to clinically affected areas twice a day. The children were reassessed on days 3 and 7 of the study. The presence of diaper rash was noted daily using a six-point scale, and in both groups, was not significantly different after the topical application of either tested medication.HBM was as effective and safe as hydrocortisone 1% ointment alone (*p* < 0.001).

In the randomized controlled trials by Seifi et al., 30 Iranian infants (between 0 and 12 months of age) suffering from diaper dermatitis were divided into two matched groups: One applying their mother’s milk three times a day to the affected area, and a control without any application for five days. The findings revealed positive effects of human milk on the healing of diaper dermatitis and a significant difference between both groups. Out of 15 infants with mild or moderate erythema, 80% improved during the five-day study, whereas in the control group 26.1% infants showed improvement (*p* = 0.009) [9].

Gozen et al. tested the effectiveness of human milk and barrier cream (40% zinc oxide with cod liver oil formulation) on the healing of diaper dermatitis. The population of the trial included 63 term and preterm newborns with developed diaper dermatitis in neonatal intensive care units, divided into two groups. There were no statistically significant differences between the groups in terms of the mean number of clinical improvement days, but the postlesion score in the barrier cream group was lower than in human milk group (*p* = 0.002) [8]. As the researchers stated, neonatal intensive care units typically host infants with disorders and who are on antibiotics. Hence, negative findings can be difficult to discuss and compare with other study data. 

### 3.2. Nipple Problems

A common breastfeeding difficulty for mothers is painful nipples. One traditional non-pharmacological intervention to reduce nipple pain in breastfeeding women is topical treatment with expressed breast milk. Abou-Dakn et al. carried out clinical trials to evaluate the efficacy of lanolin versus breast milk on painful and damaged nipples during lactation [10]. They evaluated 84 lactating mothers from Berlin who developed nipple pain while breastfeeding within 72h after delivery. The first group was instructed after each feed to express a few drops of breast milk and massage them into the nipples and areola, allowing to air-dry. The second group patted the nipples dry after each feeding session and applied a pea-sized amount of lanolin to the nipple and areola, keeping this area covered. During a number of visits over two weeks, the nipple trauma score was used to evaluate healing rates and the visual analog scale (VAS) was employed to judge the pain intensity. Significantly lower pain levels were detected in the lanolin group, and these decreased with the continuation of treatment. Lanolin was more effective than HBM, including faster healing of nipple trauma (*p* = 0.043). According to many studies, the women who applied expressed breast milk had significantly lower perceptions of nipple pain following four to five days of treatment than the women who applied lanolin. However, this beneficial effect was not maintained after six to seven days of treatment. At no assessment were there any group differences in nipple pain perceptions between the women who applied expressed breast milk and the women who had applied lanolin, warm compresses, or nothing [22].

### 3.3. Eye Problems

Treatment of ocular surface disease with human milk is documented in ancient Egyptian, Roman, Greek, and Byzantine texts and was traditionally used by mothers to treat infectious conjunctivitis. Ghaemi et al. have shown the beneficial preventive effects of colostrum against neonatal conjunctivitis on 89 breastfeeding neonates. The 269 preterm neonates were randomly divided into three groups: The first (*n* = 89) received two drops of colostrum in each eye, the second (*n* = 82) was treated with topical erythromycin ointment (0.5%), and the control group (97) received no treatment. The frequency of conjunctivitis was higher in the control group, followed by the group receiving topical application of colostrum and antibiotic (*p* = 0.003) [14]. However, colostrum does not have potential hazards or side effects and is easily accessible without extra cost. Diego et al. observed that human milk was able to preserve corneal epithelial thickness in the dry-eye mouse model. Epithelial damage, reflected in the punctate scores, decreased over four days of treatment with milk. This was the first study to demonstrate that human milk can preserve corneal epithelial thickness in a dry-eye model, and that preservation of corneal epithelial thickness was comparable to topical cyclosporine [17]. According to the study of Asena et al., topical human breast milk drops caused faster and better healing of central corneal epithelial defects in a mouse model than in the case of treatment with serum drops or artificial tears or in the control group (*p* < 0.001) [15]. They concluded that the rich contents of human breast milk may be an alternative to epithelial healers and artificial tears.

### 3.4. Umbilical Cord Care

Breast milk is widely reported to be used for umbilical cord care in developing countries, as evidenced by numerous publications. Since 1998, the World Health Organization (WHO) has advocated the use of dry umbilical cord care in high-resource settings but has also recommended research into the use of breast milk and colostrum in umbilical cord care [23]. There are several reports of the effectiveness of applying mother’s milk in assisting umbilical cord separation [12,24]. However, in the PubMed database between 2010 and 2018, only three studies were randomized controlled trials. All were published in peer-reviewed journals and were conducted in Iran (see Table 1).

Aghamohammadi et al. randomized 130 singletons, all mature and healthy newborns born in hospital, and compared the effect of topical application of human milk and dry cord care on umbilical cord separation time. Newborns were breastfed. Mothers were asked not to cover the cord with diapers and not to bath the child until the cord had separated. All mothers received instructions, a form for recording symptoms of infection and cord bleeding, and a form for observation on the progress of care. The human milk group dropped milk on the remaining part of the cord and the cut edge, letting it dry, three times a day for two days after separation of the cord. Two days after cord separation, a physician checked the cord. The median time of the cord separation was 150.95 ± 28.68 hours in the human milk topical application group and 180.93 ± 37.42 hours in the dry cord care group (*p* = 0.001). The median number of bleeding days after cord separation was 1.2 ± 2.33 and 3.1 ± 3.77, respectively [12].

In the study of Golshan et al., 300 healthy neonates, delivered normally or by Cesarean section, were divided into three random groups, in which ethanol, their mother’s milk, or dry cord care was applied. In the milk group, mothers washed the umbilical cord stump with their milk two times a day. Umbilical separation time in neonates of the human milk group was 6.5 ± 1.93 days, whereas in the ethanol and dry care group this was 8.94 ± 2.39 and 7.54 ± 2.37 days, respectively. The frequency of omphalitis was not significantly different between the three groups. Umbilical separation time in the human milk group was significantly shorter than in the ethanol (*p* < 0.0001) and drying groups (*p* < 0.003) [11].

In a clinical trial by Abbaszadeh et al., 162 healthy, hospital-borne neonates were assigned to two groups, where cord care was performed using human milk or chlorhexidine. Human milk was applied to the umbilical cord every twelve hours for days after separation. The shortest cord separation time was 3 days in the topical human milk group (7.14 ± 2.15), while the longest was 53 days in chlorhexidine group (13.28 ± 6.79) (*p* < 0.001) [13].

All three studies recommend topical application of mother’s milk for umbilical cord stump care, which leads to shorter cord separation time and can be used as an easy, cheap, natural, and noninvasive means of cord care.

### 3.5. The Antitumoricidal Effect of HAMLET

One example of translational medicine is the topical application of α-lactalbumin-oleic acid, a natural product from breast milk. The complex called human alpha-lactalbumin made lethal to tumor cells (HAMLET) was discovered by the Svanborg group when they were studying antiadhesive molecules in human milk [25].

HAMLET is formed during low-pH precipitation of the casein fraction, which allows for partial unfolding of the α-lactalbumin structure and binding with the fatty acid. HAMLET triggers rapid carcinoma cell detachment in vitro and in cancer patients after topical administration of the lyophilized complex. To form HAMLET, α-lactalbumin is obtained from human milk by chromatography. The partially unfold protein is subsequently bound to oleic acid on an ion-exchange matrix, and the complex is eluted with salt, purified, lyophilized, and frozen in aliquots for instillation [26].

The therapeutic efficacy of HAMLET has been demonstrated through in vitro research in animal models of glioblastoma, bladder cancer, and intestinal cancer; and in clinical studies targeting bladder cancers and skin papillomas [27,28]. Local HAMLET infusion was shown to delay the development of tumors and prolong survival in animal models of human cancer. In the study of Mossberg et al., groups of C57BL/6 mice with MB49-implanted murine bladder cancer cells were given instillations of HAMLET or phosphate buffered saline PBS for eight days. The HAMLET treated mice lacked detectable tumors more often than the controls (33% vs. 0%, *p* < 0.02) and the tumors were significantly reduced (mean score 1.9 vs. 2.5, respectively; *p* < 0.02) [18]. Puthia et al. tested whether HAMLET could be used for colon cancer therapy. Peroral HAMLET administration caused a significant reduction in the number of small intestinal tumors and in tumor size (*p* < 0.0001 for total tumor count), reduced polyps by about 58% (*p* = 0.0001), and improved survival (*p* = 0.0103) over the control group mice.

Through gene set enrichment analysis, the researchers concluded that the prophylactic and therapeutic effects of HAMLET are accompanied by well-defined series of stable changes in gene expression, affecting Wnt signaling and β-catenin, glycolysis, oxidative phosphorylation, and lipid metabolism in tumor tissue [19].

The same complex showed strong bactericidal activity against specific pathogens of the oral cavity and respiratory track, with the highest activity against the gram-positive organism *Streptococcus pneumoniae* by cell shrinkage, DNA condensation, and DNA degradation [29].

## 4. Discussion

The transfer of traditional medical knowledge is an ongoing process. It is important both for the preservation of traditional natural medicine, but also in the search for novel agents in treatment. Home remedies are generally believed to be natural ways to cure minor illnesses or conditions. They are usually cultural practices, traditions, customs, or folk remedies that have been passed down from generation to generation or from person to person. However, it should be kept in mind that there is not necessarily any medical proof that any of these treatments work, or whether they can cause more harm than good. Human milk is considered to be the gold standard in infant nutrition, providing optimal nutrients for normal growth and development. Apart from its nutritional benefits, human milk contains multiple bioactive and immunomodulatory components. The latter of these include growth and immunological factors, as well as micro-RNAs, cellular components such as leukocytes, epithelial cells, progenitor cells, and stem cells [30]. Furthermore, breast milk is also a continuous source of commensal and beneficial bacteria, including lactic acid bacteria and bifidobacteria [31,32]. The discovery of stem cells, the HAMLET complex, and probiotic bacteria in breast milk has resulted in increased interest in human breast milk as a natural medicine. The studies described here suggest safe and cost-effective non-nutritional uses of mothers’ breast milk, though further evaluation of effectively is needed. Breast milk has natural antibacterial properties, so it can be used to treat a range of skin problems, including cuts and scrapes.

Common skin problems may appear during lactation and breastfeeding, particularly affecting the nipple, areola, and breast. Some medications used in the treatment of skin conditions are unsuitable during lactation. It has been shown that expressing a few drops of milk and rubbing them gently into the sore nipples, then allowing it to dry naturally, takes advantage of the healing properties of human milk. Many studies have indicated that bioactive components of human milk and microbiota have promise as adjuvants for wound healing [33,34]. From lesions of the corneal epithelium to lacerations of the skin, milk-treated groups healed faster than controls.

Breast milk is used in many cultures for skin irritations. Breast milk involves no risk of allergy, contains antibodies, epidermal growth factor (EGF), and erythropoietin, which may promote the growth and repair of skin cells. Human milk is a source of commensal bacteria that can play an anti-infectious, immunomodulatory role. Their possible function in the acceleration of conditions for skin biofilm formation can open new perspectives for the prevention and treatment of skin and wound healing diseases. Interestingly, the analysis of Simpson et al. showed that miRNAs are possible mediators of the observed preventative effects of atopic dermatitis [35].

The concentration, regulation, and individual variation between bioactive element, immune factors, various progenitor and mature cell types, and stage of lactation are not well established. Complexity and variability in human milk composition, and infants’ responses to many human milk constituents may also explain some of the conflicting results of studies evaluating the effects of non-nutritional uses of human milk.

The studies considered here vary in methodology and in definition of outcomes, which leads to considerable heterogeneity. Human milk composition varies both within and between individuals, and this may partially explain the conflicting data.

However, the use of breast milk in the treatment of inflamed or injured eyes is not applicable in all cases. It should rather serve as complementary therapy, and not the only mode of treatment. At our present level of knowledge, non-nutritional uses of breast milk are certainly better suited to prevention than to a medicated process. However, in areas where mothers and infants do not have ready access to medicine, such as in developing countries, the application of breast milk is often a determining factor in infant recovery and survival.

### 4.1. Implications for Future Research

Fresh whole human milk and its components have potential as a novel therapeutic tool in the treatment of many diseases. In our opinion, the future implications of non-nutritional application are associated with particular components of breast milk, rather than with the whole milk.

In Hakansson’s study of the antimicrobial activity of human milk, a complex of α-lactalbumin and oleic acid induced apoptosis in tumor cells without affecting healthy differentiated cells. HAMLET is a tumoricidal protein–lipid complex from human milk with broad effects against cancer cells of different origins. The mechanism of its action is unusual, as the complex interacts with a number of molecular targets and cellular components. Importantly, HAMLET does not have any toxic effects on healthy tissues in the treated patients and animals. HAMLET has been shown to be safe and effective in humans in two proof-of-concept human clinical trials: Convincing therapeutic efficacy was demonstrated in a topical skin papilloma study and in patients with bladder cancer (ClinicalTrials.gov Identifier: NCT03560479) [33]. Publications on HAMLET are related to the establishment of a Swedish company, HAMLET Pharma, and the increased number of patents regarding this molecule. Based on these discoveries, HAMLET Pharma is developing natural tumor-killing drugs based on molecules with tumor selectivity. A HAMLET patent portfolio has been established with a number of patents issued worldwide. Intellectual property rights include patents covering the manufacturing and use of HAMLET and substances derived from HAMLET “second generation drug candidates”.

Many studies highlight progenitor and breast milk stem cells. The presence of stem cells in human milk poses numerous questions and implications for breastfeeding, newborn, and maternal health, but also opens a new perspective of future potential applications of these cells in personal and regenerative medicine. The goals of future research should be to assess the function, potency, and therapeutic value of breast milk cells—including cell therapy for future applications—and should determine the direct or indirect effects of breast milk cell components on promoting immunological tolerance and newborn development, and also on providing effective and complementary treatment of diaper rash, atopic eczema, diaper dermatitis, umbilical cord separation, and eye problems. Without any doubt, future research on these topics will need to involve evidence-based medicine and clinical trials.

### 4.2. Limitations

Due to the limited and heterogeneous body of evidence that included animal studies, human intervention studies, and observational human studies, the risk of bias assessment for individual studies was not performed. The overall body of evidence was narratively discussed.

This article is limited by its emphasis on papers published in English in journal databases, so many useful local ethnomedical studies may have been missed. Evidence-based medicine focuses more on new approaches than on developing traditional folk and ethnobiological data, so many promising intervention studies are not published in papers indexed in PubMed with high impact factor. It is unclear whether a lack of institutional support and funding for clinical trials of natural products might be critical in the low number of studies.

### 4.3. Conclusions

The findings of this review provide information about possible non-nutritional uses of breast milk in postnatal care. Breast milk is a natural agent and is biologically suitable for the body, having no side effects; it is always available and can be used in all social and economic groups of society. The health implications of milk components—such as macronutrients, biologically active factors, and somatic cells—remain unknown or not well understood. The positive effects of HBM found by in vitro and animal studies must be substantiated by findings from clinical studies. The most reliable clinical studies for assessing the benefits of HBM are randomized, double-blinded, multicenter controlled trials but to date, they are very scarce.

Non-nutritional uses of human breast milk can be considered examples of personalized medicine. Further research including developed countries is recommended to find or confirm the results and to evaluate the effects of traditional therapies. This knowledge may also have the effect of convincing mothers to continue to breastfeed with their own milk, as a substance that possesses extraordinary properties, not only for nutrition.

## Figures and Tables

**Table 1:** Articles included in this review, with study details.

References	Intervention/Study Type	Sample Size	The Dropout Rate	Study Design	Outcomes	Main Findings
Berents et al. (2015) [5]	Atopic eczema/Randomized clinical trial	18 participants	33%	HBM or emollient was applied on the spot, three times a day for four weeks. The severity and area of the eczema spots was calculated weekly by SCORAD.	Both control and intervention areas of the eczema spots were increased during the intervention. At inclusion mean SCORAD (SCORing Atopic Dermatitis) was 35 and at the end of study 34.	No effect with topical application of HBM was found.
Kasrae et al. (2015) [6]	Atopic eczema/Randomized clinical trial	116 participants	10%	HBM or hydrocortisone 1% was applied twice a day for 21 days on the affected area. Efficiency of the treatment was defined by SCORAD index.	The frequency of healed infants was 81.5% and 76% in HBM and 1% hydrocortisone groups on day 21, respectively (*p* < 0.24).	HBM was as effective as 1% hydrocortisone.
Farahani et al. (2013) [7]	Diaper dermatitis/Randomized clinical trial	152 participants	4.6%	HBM or hydrocortisone 1% was applied for 7 days on the affected area. The efficiency of the treatment was evaluated at 3 and 7 days by a 6-point scale.	The severity score was not different between the topical HBM and hydrocortisone 1% groups at 3 and 7 days (*p* < 0.95).	HBM was as effective as 1% hydrocortisone.
Gozen et al. (2014) [8]	Diaper dermatitis/Randomized clinical trial	70 participants	10%	HBM and barrier cream containing 40% zinc oxide and cod liver oil was applied on diaper dermatitis change for 5 days and the postlesion score was establish by a 4-point scale.	The condition of dermatitis was improved in 60% of infant from HBM group and 93.6% treated with barrier cream. The postlesion score of barrier cream group was lower than HBM group (*p* = 0.002).	Barrier cream was more effective than HBM.
Seifi et al. (2017) [9]	Diaper dermatitis/Randomized clinical trial	30 participants	0	Infants suffering from diaper dermatitis assigned to HBM group and the control group were followed up for 5 days and the efficiency of the treatment was evaluated by a 5-point scale rash severity.	In the control group 26.1% infants showed improvement, in HBM group—80%. HBM has decreased the incidence of anal dermatitis rash (*p* = 0.009).	A positive effect with topical application of HBM was found.
Abou-Dakn et al. (2011) [10]	Painful and damaged nipples/No full randomized clinical trial	84 participants	14%	The efficacy of HBM and lanolin on pain and damage nipples was assessed on the 10-range Visual Analog Scale (VAS) and the Nipple Trauma Score (NTS) over 14 days after delivery.	Lanolin was more effective than HBM, including faster healing of nipple trauma and reducing nipple pain (*p* = 0.043).	No positive effect with topical application of HBM was found.
Golshan and Hossein (2013) [11]	Umbilical cord care/Randomized clinical trial	316 participants	5%	The neonates were divided into three groups: Topical ethanol or HBM application twice a day, the control group kept the stump dry. Umbilical separation time and local infection frequency were considered.	Umbilical separation time in human milk group was significantly shorter (6.5 days) than in ethanol (8.94 days) (*p* < 0.0001) and drying groups (7.54 days) (*p* < 0.003).	A positive effect with topical application of HBM was found.
Aghamohammadi et al. (2012) [12]	Umbilical cord care/Randomized clinical trial	152 participants	14.5%	The umbilical separation time was compared in the group of topical HBM application (three times a day) and dry cord care for 10 days.	Median time of cord separation in human milk application group (150.95 ± 28.68 h) was significantly shorter than dry cord care group (180.93 ± 37.42 h) (*p* < 0.001).	A positive effect with topical application of HBM was found.
Abbaszadeh et al. (2016) [13]	Umbilical cord care/Randomized clinical trial	174 participants	6.9%	The infant from HBM group received topical application of milk and group 2 chlorhexidine solution 4% to the umbilical stump 2 times a day. Follow-up and visit at home were done.	The mean cordseparation time in the human milk group (7.14 ± 2.15 days) was shorter than the chlorhexidine group (13.28 ± 6.79 days) (*p* < 0.001).	A positive effect with topical application of HBM was found.
Ghaemi et al. (2014) [14]	Neonatal conjunctivitis/Randomized clinical trial	300 preterm neonates	10.6%	The intervention group with culture-negative eye swab received two drops of HBM in each eye or erythromycin ointment (0.5%), control group—no treatment. All neonates were followed for the occurrence of clinical conjunctivitis for 28 days.	The beneficial preventive effects of colostrum against neonatal conjunctivitis (*p* = 0.036).	A positive effect with application of HBM was found.
Asena et al. (2017) [15]	Corneal epithelial wound/Randomized trial on mice model	24 female experimental corneal epithelial defect mice model	0	A central corneal epithelial defect was created in mice and HBM, autologous serum, artificial tears four times a day was applied for 3 days. Histopathological and electron microscopy examination was performed.	Topical human breast milk drops causedfaster and better healing of central corneal epithelial defect than serum drops, artificial tears and in the control group (*p* < 0.001).	A positive effect with application of HBM was found.
Beynham et al. (2013) [16]	Antimicrobial effect on pediatric conjunctivitis/in vitro study	milk from 23 women/9 bacterial species tested	not applicable	The inhibitory effects of HBM against three common ocular pathogens were assessed. Zones of inhibition by milk samples, sterile saline, and trimethoprim ophthalmic solution were measured	Growth of *N gonorrhoeae*, *M catarrhalis*, *M viridans* was significantly inhibited (*p* ≤ 0.01) by human milk samples.	A positive effect with application of HBM was found.
Diego et al. (2016) [17]	Dry eye syndrome/Animal in vivo study	91 BALB/c mice	0	The animals with dry eye syndrome were treated with HBM, nopal, nopal extract derivatives, or cyclosporine four times daily for 7 days. Punctate staining and preservation of corneal epithelial thickness were used as indices of therapeutic efficacy.	Reduction in corneal epithelial thickness was largely prevented by administration of HBM (33.2 ± 2.5 μm).	HBM decreased epithelial damage.
Mossberg et al. (2010) [18]	Bladder cancer treatment/animal model and in vitro studies	6 C57BL mice bladder cancer model	0	Bladder tumors cells and bladder mice cancel models were instilled by HAMLET. Effects of HAMLET on tumor size and apoptosis were analyzed.	HAMLET caused a dose dependent decrease in MB49 cell viability in vitro. Five intravesical HAMLET instillations significantly decreased tumor size anddelayed development in vivo compared to controls.	HAMLET from HBM delays bladder cancer development.
Puthia et al. (2014) [19]	Colon cancer prevention and treatment/animal model and in vitro studies	Apc^Min/+^ mice colorectal tumors model	0	HAMLET was given in therapeutic and prophylactic regimens. Tumor burden and animal survival were compared, and biochemistry and molecular methods were used to determine effects on colon cancer cells.	Peroral HAMLET administration reduced tumor progression and mortality in Apc^Min/+^ mice.	HAMLET from HBM delays colon cancer development.

HBM: Human breast milk; HAMLET: human alpha-lactalbumin made lethal to tumor cells.
